# Neurocircuit dynamics of arbitration between decision-making strategies across obsessive-compulsive and related disorders

**DOI:** 10.1016/j.nicl.2022.103073

**Published:** 2022-06-04

**Authors:** Darsol Seok, Reza Tadayonnejad, Wan-wa Wong, Joseph O'Neill, Jeff Cockburn, Ausaf A. Bari, John P. O'Doherty, Jamie D. Feusner

**Affiliations:** a Division of Cognitive Neuroscience, Semel Institute for Neuroscience and Human Behavior, University of California, Los Angeles, 760 Westwood Plaza, Los Angeles, CA 90024, USA; b Division of Neuromodulation, Semel Institute for Neuroscience and Human Behavior, University of California, Los Angeles, 760 Westwood Plaza, Los Angeles, CA 90024, USA; c Division of Child and Adolescent Psychiatry, Semel Institute for Neuroscience and Human Behavior, University of California, Los Angeles, 760 Westwood Plaza, Los Angeles, CA 90024, USA; d Department of Neurosurgery, David Geffen School of Medicine, University of California, Los Angeles, 10833 Le Conte Ave, Los Angeles, CA 90095, USA; e Division of Humanities and Social Sciences, California Institute of Technology, Pasadena, CA, 1200 E. California Blvd., Code 228-77, Pasadena, CA 91125, USA; f Computation & Neural Systems Program, California Institute of Technology, Pasadena, CA, 1200 East California Boulevard, Pasadena, CA 91125, USA; g Centre for Addiction and Mental Health, 250 College Street, Toronto, ON M5T 1R8, Canada; h Temerty Faculty of Medicine, Department of Psychiatry, University of Toronto, 250 College Street, 8th floor, Toronto, ON M5T 1R8, Canada; i Department of Women’s and Children’s Health, The Karolinska Institute, Tomtebodavägen 18A, 171 77 Solna, Sweden

**Keywords:** Obsessive-compulsive disorder, Body dysmorphic disorder, Habitual decision-making, Arbitration circuit, Dynamic effective connectivity

## Abstract

Obsessions and compulsions are central components of obsessive–compulsive disorder (OCD) and obsessive–compulsive related disorders such as body dysmorphic disorder (BDD). Compulsive behaviours may result from an imbalance of habitual and goal-directed decision-making strategies. The relationship between these symptoms and the neural circuitry underlying habitual and goal-directed decision-making, and the arbitration between these strategies, remains unknown. This study examined resting state effective connectivity between nodes of these systems in two cohorts with obsessions and compulsions, each compared with their own corresponding healthy controls: OCD (n_OCD_ = 43; n_healthy_ = 24) and BDD (n_BDD_ = 21; n_healthy_ = 16). In individuals with OCD, the left ventrolateral prefrontal cortex, a node of the arbitration system, exhibited more inhibitory causal influence over the left posterolateral putamen, a node of the habitual system, compared with controls. Inhibitory causal influence in this connection showed a trend for a similar pattern in individuals with BDD compared with controls. Those with stronger negative connectivity had lower obsession and compulsion severity in both those with OCD and those with BDD. These relationships were not evident within the habitual or goal-directed circuits, nor were they associated with depressive or anxious symptomatology. These results suggest that abnormalities in the arbitration system may represent a shared neural phenotype across these two related disorders that is specific to obsessive–compulsive symptoms. In addition to nosological implications, these results identify potential targets for novel, circuit-specific treatments.

## Highlights

• Obsessive-compulsive and related disorders (OCRD) include OCD and BDD.
• Neural differences in decision-making arbitration may underlie OCRD symptoms.
• Resting-state effective connectivity was used to assess arbitration circuitry.
• Greater left putamen inhibition via left ventrolateral prefrontal cortex in OCRD.
• Stronger left putamen inhibition was correlated with less severe symptoms.

## 1 Introduction

The Diagnostic and Statistical Manual of Mental Disorders, 5th edition (DSM-5) delineates a category of disorders called the obsessive–compulsive and related disorders (OCRD) that includes obsessive–compulsive disorder (OCD), body dysmorphic disorder (BDD), hoarding disorder and excoriation disorder (skin-picking disorder). The central behavioural phenotypes in OCRD patients are (i) obsessions – recurrent, intrusive thoughts that cause significant distress – and (ii) compulsions – repetitive, stereotyped behaviours aimed at preventing or reducing distress. Although there is support for this grouping on the behavioural and symptom level, much less neurobiological evidence exists for this nosology. Decision-making perspectives of OCD have theorized that compulsive behaviours are the result of an imbalance of habitual and goal-directed decision-making strategies (Gillan et al., 2016, Gillan et al., 2017). Converging studies have elucidated the existence of two competing systems for controlling behaviour: (i) a goal-directed, deliberative system involving areas such as the caudate and orbitofrontal cortex (OFC) and (ii) an automated, habitual system based in areas including the supplemental motor area (SMA) and putamen (Balleine and Dickinson, 1998, Daw et al., 2005). Behavioural studies of OCD have revealed an over-reliance on habitual decision-making strategies in a variety of paradigms Gillan, Jul. (Gillan, 2011, Voon, 2015), and neural studies have identified aberrant functioning in cortico-striatal-thalamo-cortical (CSTC) circuitry, a set of circuits long associated with habit formation (Ahmari and Dougherty, 2015, Saxena and Rauch, 2000, Tadayonnejad, 2018).

Further studies have identified the existence of a separate mechanism that monitors the reliability of both the goal-directed and habitual systems and gates which system should control behaviour (Daw et al., 2005, Lee et al., 2014, O’Doherty et al., 2021). This “arbitration” system, involving the ventrolateral prefrontal cortex (vlPFC, BA45, inferior lateral prefrontal cortex in Lee et al., 2014) and frontopolar cortex (FPC, BA9/10), is hypothesized to downregulate the activity of the habitual system, reducing the influence of brain regions like the posterolateral putamen on motor systems downstream. Several functional studies corroborate this account (Daw et al., 2005, Lee et al., 2014, O’Doherty et al., 2021), and there is evidence for structural connections between the vlPFC and the putamen (Haber et al., 2021). Dysfunction in nodes of the arbitration system like the vlPFC may underlie OCRD patients’ overreliance on habitual learning and behaviour. Reduced gray matter volumes (van den Heuvel, 2008), abnormal connectivity patterns (Anticevic, 2014) and reduced activation during reversal learning (Chamberlain, 2008) in these brain regions support this possibility.

Despite emerging evidence, no studies have specifically examined to what extent abnormalities exist in the directed, causal relationships between the arbitration and habitual systems in OCRD disorders. These causal relationships can be inferred using functional magnetic resonance imaging (fMRI) data and effective connectivity analysis techniques based on, for example, Granger causality (Roebroeck et al., 2005, Wen et al., 2013) or dynamic causal modeling (Friston, 2009). Given that the arbitration system operates by dynamically downregulating the influence of the habitual system on motor systems, a less inhibitory (weaker negative effective connectivity) or less dynamic (less variable connectivity) arbitration system may result in the stereotyped and inflexible reliance on habitual behaviours that is characteristic of OCRD symptoms. Additionally, within OCRD individuals, those with more inhibitory arbitration systems may be more capable of suppressing habitual systems and therefore exhibit less severe symptomatology. Alternatively (or in addition to abnormalities in the arbitration system), exaggerated connectivity within the habitual system or impoverished connectivity within the goal-directed system may underlie these symptoms.

It remains unclear if dysfunction of these neural systems could represent a *shared* phenotype across different OCRDs. Additionally, because anxiety and depression symptoms are commonly present in individuals with these disorders, an important consideration is whether any identified neural abnormalities are *specific* to OCRD symptoms. Answering these questions will contribute to the refinement of the field’s neurobiological and nosological understanding of these conditions, including their shared and distinct etiology.

To this end, this study examines resting state effective connectivity in two cohorts of individuals with OCRD: OCD and BDD. Based on regions of interest (ROIs) identified in previous studies (Lee et al., 2014), we examined the magnitude and variability of three directed connections between the arbitration and habitual systems: left vlPFC → left posterolateral putamen, right vlPFC → left posterolateral putamen, right FPC → right posterolateral putamen. Per our preregistered hypotheses (https://aspredicted.org/675hy.pdf, https://aspredicted.org/w8hj7.pdf) we predicted that: (i) these connections would be significantly attenuated and less variable relative to controls; (ii) within OCRD participants, stronger inhibitory connectivity would be associated with lower symptom severity. We also predicted that these associations would be specifically associated with obsessive–compulsive severity and not symptoms of depression or anxiety. Additional exploratory analyses tested for abnormalities within the habitual system (SMA → posterolateral putamen, one connection per hemisphere) and within the goal-directed system (left/right caudate → OFC).

## 2 Materials and methods

### 2.1 Participants

Two sets of data, collected independently, one of OCD participants (Feusner, 2015) and another of BDD participants, were analyzed. All participants were recruited from University of California, Los Angeles (UCLA) clinics and in the community through flyers and online advertisements. Additionally, both sets of data included separate healthy control groups that had equivalent age and gender characteristics as their respective clinical samples.

The OCD participants were those who met eligibility criteria, including a primary diagnosis of OCD. The BDD participants were those who met eligibility criteria, including a primary diagnosis of BDD. OCD participants could be unmedicated or medicated, provided they had no changes in medication for at least 12 weeks prior to recruitment. BDD participants currently on psychotropic medications were excluded. For full inclusion/exclusion criteria, please see the Supplementary Materials.

### 2.2 Clinical assessments

Obsessive compulsive symptomology in the OCD group was assessed using the YBOCS (Goodman, 1989). BDD participants were assessed using a version of the YBOCS modified for BDD, the BDD-YBOCS (Phillips et al., 1997). The two additional questions in the BDD-YBOCS related to insight and avoidance were not included, to correctly compare results across two clinical groups.

Depressive symptomology was measured using the Montgomery-Asberg Depression Rating Scale (MADRS; (Montgomery and Åsberg, 1979). Anxiety symptomology was measured using the Hamilton Anxiety Rating Scale (HAM-A; (Hamilton, 1959)).

### 2.3 Image acquisition

For both datasets, resting state fMRI data was collected on a 3 T Siemens Trio using a T2*-weighted echo planar imaging (EPI) sequence. Pulse sequences between these datasets differed, most significantly in TR (2000 ms for OCD, 720 ms for BDD), which has been shown to affect estimates of effective connectivity (Witt and Meyerand, 2009). Therefore, analyses were conducted separately for these two groups. Please see the Supplemental Materials for all imaging parameters.

### 2.4 Image preprocessing

Structural and functional images from both datasets were processed using FMRIPREP (Esteban, 2019). We included slice timing correction and denoising with ICA-based Automatic Removal Of Motion Artifacts (for full details, see Supplementary Materials).

### 2.5 Dynamic effective connectivity (DEC)

Multiple fMRI (Rangaprakash, 2018, Wu et al., 2013), simulation (Wen et al., 2013, Ryali et al., 2011, Deshpande et al., 2009) and experimental studies utilizing electrophysiology and optogenetics (Katwal et al., 2013, David, 2008, Ryali, 2016, Wang et al., 2017) support the utility of deconvolving the hemodynamic response function (HRF) from fMRI time series prior to estimating effective connectivity. This step minimizes of the effect of inter-individual and inter-regional variability of the HRF and further improves accuracy of effective connectivity estimates. Therefore, prior to computing measures of DEC, we performed voxel-wise, blind-source hemodynamic deconvolution (Wu et al., 2013, Havlicek et al., 2011) using rsHRF (https://www.nitrc.org/projects/rshrf).

Deconvolved timeseries were sampled from key connections of the habitual, arbitration and goal-directed systems (Lee et al., 2014, Wunderlich et al., 2012), using spherical ROIs of radius 5 mm centered on the following MNI coordinates: L vlPFC (−54, 38, 3), R vlPFC (48, 35, −2), R FPC (15, 56, 25), L posterolateral putamen A (−27, −19, 4), L posterolateral putamen B (−36, −22, −8), R posterolateral putamen (33, −10, 1), L SMA (−9, 8, 55), R SMA (9, 8, 55), L caudate (−9, 15, 3), R caudate (9, 15, 3) and OFC (−3, 38, −11) (two separate seeds were used for the L posterolateral putamen following the results of Lee et al. (2014).

Finally, measures of DEC were estimated by employing Kalman-filter based time-varying Granger causality on the timeseries (Büchel and Friston, 1998, Tadayonnejad, 2016, Tadayonnejad et al., 2016). Effective connectivity estimates the directed causal influence of one brain region on another brain region. Negative effective connectivity from one brain region to another may indicate an inhibitory influence of one brain region on another while positive effective connectivity may indicate an excitatory influence (Hamilton et al., 2011). Deconvolved timeseries from each ROI were inputs to a dynamic multivariate autoregressive (dMVAR) model, which was solved in a Kalman-filter framework. The results are 34 DEC timeseries for each participant, one for each of the possible directed connections.

**Fig. 1:**
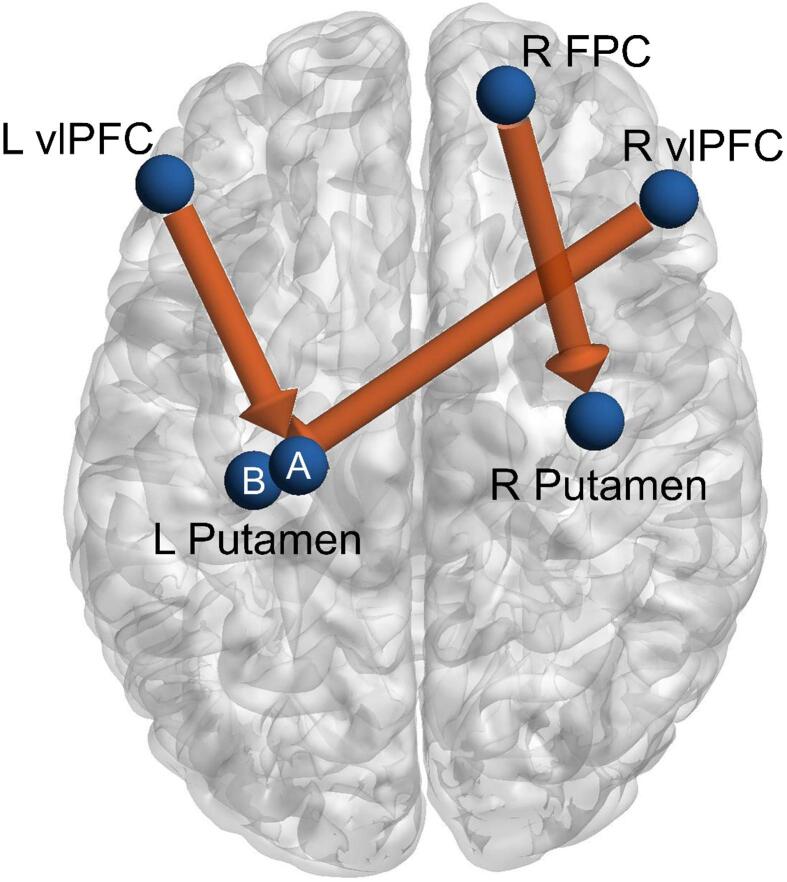
Schematic diagram of the three arbitration connections assessed. Arrows indicate directionality of dynamic effective connectivity. Two seeds were used for the left posterolateral putamen (“L Putamen”): one (“A”) connecting to the left ventrolateral prefrontal cortex (“L vlPFC”) and the other (“B”) to the right ventrolateral prefrontal cortex (“R vlPFC”). R Putamen = right posterolateral putamen, R FPC = right frontal polar cortex.

We analyzed three directed connections in the arbitration system: L vlPFC → L posterolateral putamen A, R vlPFC → L posterolateral putamen B, R FPC → R posterolateral putamen (Fig. 1). These connections were selected as they previously have been shown to exhibit significant modulation as a result of changing reliability of the habitual system (indicating the activity of the arbitration system) (Lee et al., 2014). Additionally, we examined connections *within* the habitual (L SMA → L posterolateral putamen A, R SMA → R posterolateral putamen) and *within* the goal-directed systems (L caudate → OFC, R caudate → OFC). The results of analyses for the remaining 27 directed connections are included in Supplemental Tables 2 and 4. The direction/magnitude and variability of connections were summarized by computing the mean and standard deviation, respectively.

### 2.6 Statistical modeling

Omnibus group differences in the mean and variability of DEC were assessed using a one-factor MANCOVA that included the three directed arbitration connections as the dependent variables, group as the independent variable and age, gender, and motion (spatial standard deviation of successive difference images, or “DVARS”) as covariates. Separate tests were performed for the mean and variability of DEC. Separate MANCOVAs were also conducted for the within-habitual and within-goal-directed connections.

Separate models were fit for the OCD and BDD datasets. If a significant group difference was detected, post-hoc linear regressions examining group differences were fit, one for each connection. *P-*values for post-hoc models were subject to Bonferroni correction. Cohen’s *d* was also computed to compare effect sizes across the two datasets.

Associations between DEC measures and obsessive–compulsive symptomology (YBOCS) were examined using multiple linear regression, including all three arbitration connections as regressors. As a follow-up, *p*-values for each connection were Bonferroni corrected for the three arbitration connections. Separate models were also fit for the within-habitual and within-goal-directed connections. Pearson correlation coefficients for significantly associated connections were computed to allow us to compare effect sizes across the two datasets. We also examined associations with MADRS and HAM-A scores.

As a post-hoc analysis, we explored potential moderating effects of psychotropic medication in symptom-connectivity relationships. For this, we tested a linear model that included an interaction term between DEC measures and medication status (expressed as a binary variable: medicated with any psychotropic medications OR unmedicated). We generated a model for every connection that exhibited significant association with symptom severity. These analyses were only conducted for the OCD dataset, since all BDD participants were unmedicated.

### 2.7 Preregistration

Hypotheses and methods for the regions/connections of interest were preregistered through aspredicted.org (https://aspredicted.org/675hy.pdf and https://aspredicted.org/w8hj7.pdf) prior to analysis. Exploratory analyses of the within-habitual and within-goal-directed connections and the post-hoc analysis of medication effects were not preregistered. All code for preprocessing and analysis is available on GitHub (https://github.com/dseok/ocd_bdd_arbitration_rsfmri).

## 3 Results

For the OCD dataset, 45 OCD participants and 25 healthy controls were enrolled. One participant was not included in the analyses due to a change in medication status immediately prior to the study. Additionally, we excluded one OCD participant’s and one healthy control’s data due to excessive head motion (DVARS > two standard deviations of the mean), leaving 43 OCD and 24 healthy control participants for the OCD dataset.

For the BDD dataset, 24 BDD participants and 20 healthy controls were recruited. Three BDD and three healthy control participants were excluded due to excessive head motion and one healthy participant due to poor image registration to template space. This left 21 BDD participants and 16 healthy controls for the BDD dataset.

Demographic information for all analyzed participants is presented in Table 1. OCD participants and BDD participants did not significantly differ in age or gender with their respective healthy controls. Additionally, OCD and BDD participants did not significantly differ in their degree of obsessions and compulsions (*p* > 0.15), depression (*p* > 0.8) or anxiety (*p* > 0.4).

**Table 1:** Demographic and psychometric information. Each patient group was recruited with its own matched sample of healthy controls, and the patient group associated with each group of healthy controls (HC) is indicated in parentheses. Displayed are test statistics and *p* values for two-sample *t* tests or chi-squared tests (gender) comparing OCRD groups with their respective healthy controls. OCD = obsessive compulsive disorder, BDD = body dysmorphic disorder, YBOCS = Yale-Brown Obsessive Compulsive Scale. Note that for participants with body dysmorphic disorder we used only items 1–10 of the BDD-YBOCS (YBOCS modified for body dysmorphic disorder) to match the structure of the OCD YBOCS. See Methods for further details.

	OCD	HC (OCD)	*t* or χ^2^ (df)	*p*	BDD	HC (BDD)	*t* or χ^2^ (df)	*p*
N	43	24	–	–	21	16	–	–
Age (SD)	33.05 (10.67)	30.96 (11.98)	0.735 (65)	0.465	22.38 (4.40)	22.63 (6.84)	−0.132 (35)	0.896
# female	21	10	0.095 (1)	0.757	18	12	0.161 (1)	0.689
# medicated	14	0	–	–	0	0	–	–
YBOCS or BDD-YBOCS (SD)	24.51 (4.68)	–	–	–	22.81 (4.04)	–	–	–
MADRS (SD)	15.28 (9.49)	1.13 (1.23)	7.247 (65)	<0.001	14.71 (9.84)	1.19 (1.52)	5.430 (35)	<0.001
HAM-A (SD)	12.42 (5.35)	1.38 (1.21)	9.939 (65)	<0.001	11.05 (8.16)	2.69 (2.21)	3.974 (35)	<0.001

Please see Supplemental Table 1 for a list of comorbidities in each group. Two OCD participants had comorbid BDD (although OCD was their primary diagnosis), and no BDD participants had comorbid OCD.

### 3.1 Group comparisons

**Fig. 2:**
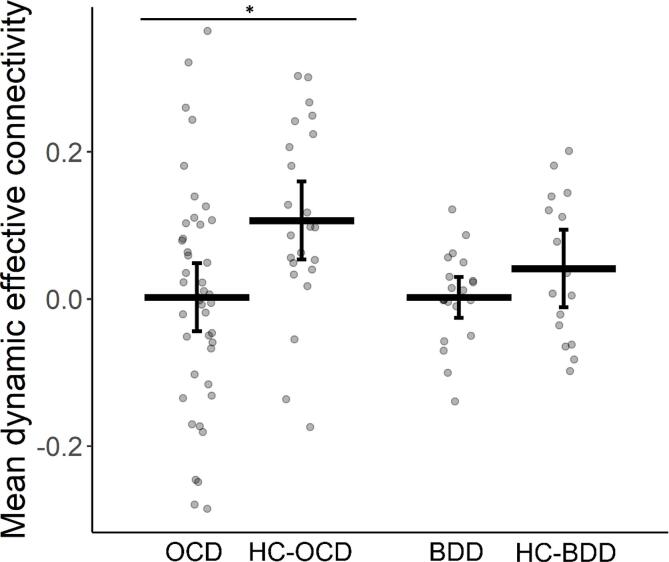
Group differences in the mean dynamic effective connectivity for the left ventrolateral prefrontal cortex → left posterolateral putamen connection. Each dot represents a single participant, thick horizontal lines represent group means and brackets indicate 95% confidence intervals. *Indicates a significant difference between patients and controls (*p*_corrected_ < 0.05). OCD = obsessive–compulsive disorder, HC-OCD = healthy controls from the OCD dataset, BDD = body dysmorphic disorder, HC-BDD = healthy controls from the BDD dataset.

MANCOVA analyses detected a significant difference in mean DEC for connections involving the arbitration system between OCD participants and healthy controls (Pillai’s Trace = 0.126, *F*(3, 60) = 2.880, *p* = 0.043). Post-hoc analyses revealed that mean DEC for the L vlPFC → L posterolateral putamen connection was significantly more negative in OCD participants compared to healthy controls (Fig. 2; *β_group_* = -0.109, *t*(62) = -2.908, *p_corrected_* = 0.015, Cohen’s *d* [95% CI] = 0.74 [0.23, 1.26]). OCD participants and healthy controls did not significantly differ in DEC variability (*p* > 0.5). Neither MANCOVA for the within-habitual (*p* > 0.7) nor within-goal-directed connections (*p* > 0.7) was significant.

The MANCOVA revealed no significant group differences in mean DEC (*p* > 0.25) or DEC variability (*p* > 0.5) for the arbitration system connections in the BDD dataset. However, exploring the mean DEC of the L vlPFC → L posterolateral putamen connection, there was a group difference trending in the same direction as in OCD dataset (Fig. 2; *β_group_* = -0.045, *t*(32) = -1.595, *p_uncorrected_* = 0.121, Cohen’s *d* [95% CI] = 0.53 [−0.13, 1.19]). A post-hoc power analysis for this connection suggested that the BDD dataset (*n* = 37) was underpowered to detect a group difference of the same magnitude as the OCD dataset (*n* = 48 required for 85% power for one-sided test). As in OCD, neither MANCOVA for the within-habitual (*p* > 0.75) nor within-goal-directed connections (*p* > 0.7) was significant.

Complete results for all connections in the OCD and BDD datasets are presented in Supplemental Table 2.

### 3.2 Associations with obsessive–compulsive symptom severity

**Fig. 3:**
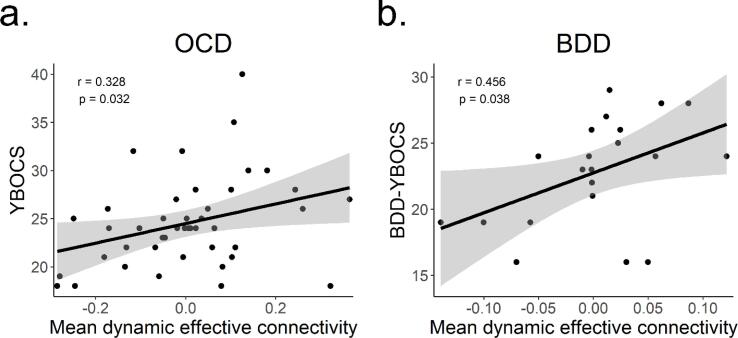
Correlations between mean dynamic effective connectivity in the left ventrolateral prefrontal cortex → left posterolateral putamen connection and YBOCS and BDD-YBOCS. *a*. Results for the obsessive–compulsive disorder dataset. *b*. Results for the body dysmorphic disorder dataset. Shaded areas indicate 95% confidence intervals. OCD = obsessive compulsive disorder, BDD = body dysmorphic disorder, YBOCS = Yale-Brown Obsessive Compulsive Scale, BDD-YBOCS = Yale-Brown Obsessive Compulsive Scale modified for BDD.

In the OCD dataset the L vlPFC → L posterolateral putamen connection was positively associated with YBOCS (Fig. 3a; *β* = 12.157, *t*(39) = 2.561, *p_corrected_* = 0.042, Pearson correlation [95% CI] = 0.328 [0.030, 0.572]). No other connections in the arbitration set exhibited significant associations (see Supplemental Table 3 for full models). No connections in either the within-habitual (*p_corrected_* > 0.4) or within-goal-directed (*p_corrected_* > 0.2) connections exhibited significant associations. DEC variability across all connections was not significantly associated with YBOCS.

In BDD, no connections were significantly associated with the BDD-YBOCS. However, the L vlPFC → L posterolateral putamen connection showed a (nonsignificant) positive association in the same direction as in the OCD dataset (*β* = 23.542, *t*(17) = 1.583, *p_uncorrected_* = 0.132). The bivariate correlation between this connection and YBOCS was positive (Pearson correlation [95% CI] = 0.456 [0.030 0.742], *p_uncorrected_* = 0.038; Fig. 3b). For completeness, bivariate correlations between mean DEC and BDD-YBOCS for all connections in the OCD and BDD datasets are presented in Supplemental Table 4. As in the OCD dataset, DEC variability across all connections was not significantly associated with BDD-YBOCS.

MADRS scores were not significant correlated with mean DEC in the L vlPFC → L posterolateral putamen connection in either the OCD (Pearson correlation [95% CI] = 0.198 [−0.109 0.471], *p* = 0.203) or BDD (Pearson correlation [95% CI] = 0.242 [−0.212 0.610], *p* = 0.292) datasets. Similarly, HAM-A scores were not significantly correlated with mean DEC in this connection in either the OCD (Pearson correlation [95% CI] = 0.221 [−0.085 0.489], *p* = 0.155) or BDD (Pearson correlation [95% CI] = 0.230 [−0.223 0.602], *p* = 0.315) datasets.

**Fig. 4:**
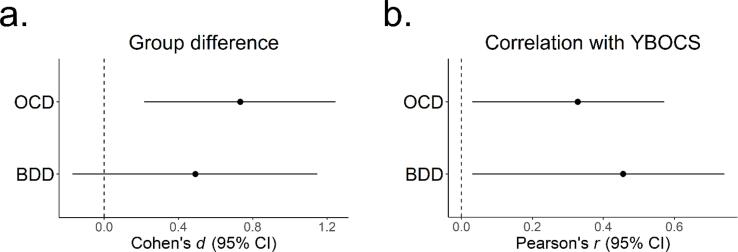
Forest plots of effect sizes for mean dynamic effective connectivity (DEC) of the left ventrolateral prefrontal cortex → left posterolateral putamen connection for each dataset. Plotted are point estimates of effect sizes and 95% confidence intervals. *a.* Cohen’s *d* for a group difference in mean DEC between each patient group and their associated sample of healthy controls. *b.* Pearson’s *r* between mean DEC and Yale-Brown Obsessive Compulsive Scale (YBOCS) for OCD and the BDD-YBOCS for BDD. OCD = obsessive compulsive disorder, BDD = body dysmorphic disorder.

Effect sizes for group differences and associations with YBOCS (OCD) and BDD-YBOCS (BDD) for both datasets are shown in Fig. 4.

### 3.3 Medication effects in OCD

There was a significant interaction between medication status and mean DEC of the L vlPFC → L posterolateral putamen connection (β = 21.049, t(39) = 2.300, p = 0.027; Supplemental Fig. 1). Medicated participants exhibited a positive relationship (Pearson correlation [95% CI] = 0.608 [0.115 0.861], *p* = 0.021), whereas unmedicated participants did not (Pearson correlation [95% CI] = 0.094 [−0.282 0.445], *p* = 0.628).

## 4 Discussion

The goals of this study were to investigate associations between the neural substrates of decision-making and obsessive-compulsion related symptomatology across OCD and BDD. Within arbitration circuitry, the L vlPFC → L posterolateral putamen connection exhibited more negative effective connectivity compared to healthy controls as well as positive associations with obsessive–compulsive symptom severity. These results suggest that abnormalities in effective connectivity between the arbitration and habitual systems may represent a common neural phenotype across these two OCRD disorders. Importantly, this association may be specific to both the arbitration system and OCRD symptoms: connectivity within the habitual and goal-directed systems was not significantly associated with group or OCRD symptom severity, and the L vlPFC → L posterolateral putamen connection was not associated with either depression or anxiety symptoms.

While we predicted that OCRD participants would exhibit an attenuated (less inhibitory) arbitration system, we observed the opposite: OCD participants exhibited more negative DEC in the L vlPFC → L posterolateral putamen compared to controls, and BDD participants had a trend in the same direction (non-significance may be due to smaller sample size given the larger effect size observed or may reflect a null result). We observed that, in the resting state, healthy controls generally exhibited positive DEC while the mean of OCD and BDD participants was closer to zero, which seems contrary to previous task-based fMRI results identifying an inhibitory connection (Lee et al., 2014). However, in the absence of an overt task, it is possible for connections to change their DEC magnitudes and even reverse sign (Li et al., 2012). Therefore, this connection might only become absolutely negative in sign during a relevant decision-masking task. Nonetheless, given that resting state connectivity patterns are theorized to reflect longstanding regional brain interactions that are typically engaged in day-to-day life outside of the scanner (Tsodyks, 1999, Lewis et al., 2009), the *relative* difference between healthy controls and OCRD participants may be a key finding. This result could reflect that individuals with OCRD may experience greater tonic load on their arbitration system, which needs to exert more frequent inhibitory control to compensate for a hyperactive habitual system. Alternatively, the results could represent a state effect in which OCRD individuals may be more likely than controls to engage their arbitration system *within* the scanner to resist urges to perform compulsions. The latter explanation would appear less likely due to the heterogenous nature of OCD symptoms such that some individuals experience spontaneous intrusive thoughts and urges to perform compulsions whereas for others this occurs primarily when being triggered by external stimuli. Larger studies, especially those studying individuals with BDD, are necessary to confirm this finding.

As predicted, stronger inhibitory connectivity between the arbitration and habitual systems was associated with less severe obsessive–compulsive symptoms in both OCD and BDD groups, suggesting that utilization of the arbitration system may be instrumental in successful resisting of compulsive behaviours. Further, confidence intervals for this association were highly overlapping across OCD and BDD, suggesting that the strength of this effect is similar across these two populations. It should be noted, however, that the confidence intervals for both estimates are large, which may be due to limited sample size. Effective connectivity in this connection was not significantly associated with depressive or anxious symptomology, highlighting the potential specificity of this association with obsessive–compulsive symptoms. However, given the sample sizes, non-significance of associations of connectivity with these symptoms is weak support for specificity. Future larger studies are necessary to confirm the generalizability and specificity of these phenotypes.

Connectivity strengths within the habitual and goal-directed systems were neither significantly different between OCRD groups and healthy controls nor significantly associated with OCRD symptom severity. Thus, the findings in the OCD and BDD groups in the arbitration system suggest that abnormalities in this system may be a primary feature of OCRD pathophysiology. However, non-significance does not necessarily imply that the habitual and goal-directed systems are not implicated in the pathophysiology of OCRD, particularly given the small set of connections (two connections each) tested within these systems and the fact that this study examined connections in the resting state rather than during a task.

A previous study in OCD provided evidence that deficits in goal-directed planning and general cognitive control may underlie OCD psychopathology and symptom severity (Vaghi, 2017), as opposed to a specific overreliance on habitual decision-making. In fact, deficits in both cognitive control and the arbitration between goal-directed and habitual decision-making may be operative in OCD, particularly since there is neural evidence linking areas such as the FPC to both the arbitration system and cognitive control processes (Koechlin and Hyafil, 2007).

These findings have potential clinical implications. Given current evidence for its role in arbitration circuitry relevant to obsessions and compulsions and its accessible location on the cortex, the L vlPFC presents a prime target for neuromodulatory techniques like repetitive transcranial magnetic stimulation (rTMS) or transcranial direct current stimulation (tDCS). Previous studies in OCD have targeted other brain regions that also have roles in decision-making, such as the OFC (Ruffini et al., 2009), SMA (Gomes et al., 2012, Mantovani et al., 2010, D’Urso et al., 2016) and dorsolateral prefrontal cortex (Tadayonnejad, 2020, Najafi, 2017) but have not specifically targeted nodes of the arbitration system. Given the association between stronger inhibition of the habitual system and lower symptom severity, bolstering the L vlPFC through stimulation could augment the arbitration system’s ability to regulate the activity of the habitual system. Another possibility is to pair stimulation of this target with active behavioural interventions that involve systematic reduction in compulsions, such as cognitive-behavioural therapy (CBT) and exposure and response prevention (ERP). Such targeted neuromodulation of the vlPFC might synergistically help patients better resist these behaviours, thereby “converting” those who otherwise would not respond or have insufficient response to CBT/ERP alone.

Analyses of medication effects in the OCD dataset revealed that only medicated participants exhibited a positive relationship between effective connectivity in the left vlPFC → L posterolateral putamen connection and obsessions/compulsions. The limited sample size of medicated OCD participants (n = 14) precludes definitive conclusions about if or how medication status influences connectivity and how relationships with symptoms compare across groups. Further, because this is a cross-sectional study, we cannot make definitive conclusions about causality. For example, medication could potentially have a direct or interactive effect on connectivity or, alternatively, separate inherent factors could have contributed to one subgroup pursuing and receiving treatment with medication. Addressing the question of the medication status is challenging and will likely involve larger, longitudinal studies that are outside of the scope of the current investigation. Nonetheless, the observation of a similarly positive trend between connectivity and symptom severity in the (entirely) unmedicated BDD cohort suggests that this mechanism may have some independence from medication status.

### 4.1 Limitations and considerations

Several other limitations should be considered. Most notably, these data were collected from two separate studies; thus, differences in scanners and acquisition protocols may have affected results. For example, participants in the OCD study were instructed to keep their eyes closed during the scan while participants in the BDD study were instructed to keep their eyes open. While studies have identified differences in visual and auditory system connectivity based on eyes-open and eyes-closed protocols, none have identified significant differences in default mode or cognitive control regions (Patriat, 2013, Agcaoglu et al., 2019). Our analyses revealed comparable effect sizes across both datasets, highlighting the robustness of these associations across diverse populations and imaging parameters. Further, although the TRs differed between studies, estimates of DEC were highly correlated before and after temporal resampling (Supplemental Fig. 2, *r* range = [0.887, 0.973]), and the magnitudes of the group difference and correlation with BDD-YBOCS were comparable after resampling (Supplemental Fig. 3). Another limitation is that the BDD group was underpowered to detect similar group differences as the OCD group.

Some ROIs, particularly the left and right putamen seeds, exhibited high levels of white matter in many participants (Supplemental Fig. 4). This replicates previous studies indicating high white matter content in the putamen (Tamagaki, 2005), but potentially brings into question whether our results represent differences in “true”, neural activity between OCRD participants and healthy controls. However, a growing body of work suggests that white matter activation reflects more than physiological noise and may constitute genuine neural activity (Grajauskas et al., 1024). For example, (Wang et al., 2020) found that fluctuations in BOLD signal in white matter reflect neural activity in surrounding gray matter, and (Li et al., 2020) found that white matter voxels can exhibit consistent, significant engagement with functional networks in gray matter. Together, these results suggest that our findings represent alterations in neural activity, although further studies of corticostriatal circuitry in OCRD individuals are necessary to confirm these results.

Finally, participants were evaluated with resting-state scans, so it remains unclear whether these associations truly reflect neurobehavioral deficits in decision-making arbitration or are related to other functions of the vlPFC and posterolateral putamen. While this hypothesis-driven study selected these connections *a priori* due to their associations with decision-making processes, only future task-based studies can definitively draw the links between aberrant arbitration behaviors, abnormalities in the left vlPFC → L posterolateral putamen connection and OCRD symptoms.

### 4.2 Conclusion

This represents the first study to investigate, and provide evidence for, transdiagnostic abnormalities in the arbitration system across OCRD disorders. Heightened inhibitory influence of the arbitration system on the habitual system may operate to downregulate compulsive behaviours in both OCD and BDD. These results have nosological implications for understanding the neurobiological relationships between specific OCRDs and could potentially be utilized in future studies to test the effects of neuromodulatory interventions that target the arbitration and habit systems.

## Declaration of Competing Interest

The authors declare the following financial interests/personal relationships which may be considered as potential competing interests: JF has received consultant fees from NOCD, Inc. All other authors have nothing to disclose.
