# Supplementary material for: Neurocircuit dynamics of arbitration between decision-making strategies across obsessive-compulsive and related disorders
Source: Neuroimage Clin. 2022 Jun 4;35:103073. doi: 10.1016/j.nicl.2022.103073 (PMC9192960; doi:10.1016/j.nicl.2022.103073)
Supplement: Supplementary data 1 [file mmc1.docx]

**Supplementary Materials**

*Inclusion and Exclusion Criteria*

OCD and comorbid diagnoses were determined using the Anxiety Disorders Interview Schedule for DSM-IV-Mini (ADIS-IV-MINI; [1]). OCD participants were eligible if they scored ≥16 on the Yale-Brown Obsessive Compulsive Scale (YBOCS; [2]). Exclusion criteria included lifetime psychotic, bipolar, substance dependence, or attention-deficit hyperactivity disorders. Comorbid anxiety and depressive disorders were permitted if OCD was the primary diagnosis; however, individuals were excluded if their ADIS-IV clinical significance rating for depressive symptoms was ≥ 6 (severe).

For the BDD participants, exclusion criteria included lifetime psychotic disorders, lifetime bipolar disorder, lifetime ADHD, or current substance use disorders. The Mini International Neuropsychiatric Interview (MINI) and BDD Module [3, 4] were administered to determine BDD and comorbid diagnoses. Comorbid anxiety and depressive disorders were permitted if BDD was the primary diagnosis. Exclusionary criteria for OCD, BDD and healthy controls included suicidality, self-injurious behaviour, lifetime neurological disorders and current pregnancy.

*Imaging Parameters*

For the OCD dataset, resting state fMRI data were collected on a 3T Siemens Prisma scanner using a T2*-weighted EPI sequence (duration = 6:56 min; repetition time [TR] = 2000ms; echo time [TE] = 25ms; flip angle = 78; voxel size = 3mm isometric; 35 axial slices; 65 x 65 matrix). Participants were instructed to lie still with eyes closed and remain awake. T1-weighted structural MRI (axial magnetization-prepared rapid gradient echo [MPRAGE]; TR = 1900ms; TE = 3.26ms; voxel size = 1mm isometric) was acquired for image registration.

For the BDD dataset, resting state fMRI data was collected on a 3T Siemens Prisma scanner using a T2*-weighted EPI sequence (duration = 6:50.4 min; TR = 720ms; TE = 37ms; flip angle = 52; voxel size = 2mm isometric; 72 axial slices; 104 x 104 matrix). Participants were instructed to lie still with eyes open and to view the fixation cross on the screen. T1-weighted structural MRI [MPRAGE; TR = 2300ms; TE = 2.27ms; voxel size = 1 x 0.977 x 0.977 mm3] was acquired for registration.

*Image Preprocessing*

Results included in this manuscript come from preprocessing performed using FMRIPREP version 1.4.0 [5], a Nipype [6] based tool. Each T1w (T1-weighted) volume was corrected for INU (intensity non-uniformity) using N4BiasFieldCorrection v2.1.0 [7] and skull-stripped using antsBrainExtraction.sh v2.1.0 (using the OASIS template). Spatial normalization to the ICBM 152 Nonlinear Asymmetrical template version 2009c [8] was performed through nonlinear registration with the antsRegistration tool of ANTs v2.1.0 [9], using brain-extracted versions of both T1w volume and template. Brain tissue segmentation of cerebrospinal fluid (CSF), white-matter (WM) and gray-matter (GM) was performed on the brain-extracted T1w using fast (FSL v5.0.9, [6]).

Functional data was slice time corrected using 3dTshift from AFNI v16.2.07 [10] and motion corrected using mcflirt (FSL v5.0.9; [11]). "Fieldmap-less" distortion correction was performed by co-registering the functional image to the same-subject T1w image with intensity inverted [12, 13] constrained with an average fieldmap template [14], implemented with antsRegistration (ANTs). This was followed by co-registration to the corresponding T1w using boundary-based registration [15] with six degrees of freedom, using flirt (FSL). Motion correcting transformations, field distortion correcting warp, BOLD-to-T1w transformation and T1w-to-template (MNI) warp were concatenated and applied in a single step using antsApplyTransforms (ANTs v2.1.0) using Lanczos interpolation.

ICA-based Automatic Removal Of Motion Artifacts (AROMA) was used to generate aggressive noise regressors as well as to create a variant of data that is non-aggressively denoised [16]. Non-aggressively denoised data were used for further analyses.

Many internal operations of FMRIPREP use Nilearn [17] principally within the BOLD-processing workflow. For more details of the pipeline see <https://fmriprep.readthedocs.io/en/stable/workflows.html>.

| Disorder | OCD | BDD |
| --- | --- | --- |
| Generalized Anxiety Disorder | 8 | 5 |
| Social phobia | 7 | 4 |
| Major Depressive Disorder | 6 | 6 |
| Social Anxiety Disorder | 5 | 0 |
| Specific phobia | 5 | 0 |
| Post-Traumatic Stress Disorder | 1 | 2 |
| Social Anxiety | 1 | 0 |
| Dysthymia | 1 | 2 |
| Panic Disorder | 1 | 2 |
| Panic Disorder w/o agoraphobia | 1 | 0 |
| Depressive disorder not otherwise specified | 1 | 0 |
| Agoraphobia w/o Panic Disorder | 0 | 1 |
| Body Dysmorphic Disorder | 2 | -- |
| Obsessive Compulsive Disorder | -- | 0 |

**Supplemental Table 1.** Comorbidity information for each patient group. Listed are the number of individuals within each patient group who was diagnosed with a psychiatric comorbidity.

| *OCD* | *β_Group_* | SE | *t* | *df* | *p_uncorrected_* |
| --- | --- | --- | --- | --- | --- |
| L vlPFC → L Pu (A) | -0.109 | 0.037 | -2.908 | 62 | **0.005*** |
| R vlPFC → L Pu (B) | 0.038 | 0.044 | 0.855 | 62 | 0.396 |
| R FPC → R Pu | 0.044 | 0.041 | 1.073 | 62 | 0.288 |
| L SMA → L Pu (A) | 0.007 | 0.038 | 0.175 | 62 | 0.862 |
| R SMA → R Pu | -0.041 | 0.048 | -0.848 | 62 | 0.400 |
| L caudate → OFC | -0.038 | 0.049 | -0.770 | 62 | 0.444 |
| R caudate → OFC | 0.028 | 0.043 | 0.633 | 62 | 0.529 |
| R FPC → L Pu (A) | 0.079 | 0.038 | 2.078 | 62 | 0.042* |
| R FPC → L Pu (B) | -0.012 | 0.040 | -0.311 | 62 | 0.757 |
| R FPC → L vlPFC | -0.350 | 0.039 | -0.886 | 62 | 0.379 |
| R FPC → R vlPFC | 0.024 | 0.043 | 0.560 | 62 | 0.577 |
| L Pu (A) → R FPC | 0.071 | 0.036 | 1.990 | 62 | 0.051 |
| L Pu (A) → L Pu (B) | 0.069 | 0.035 | 1.957 | 62 | 0.055 |
| L Pu (A) → R Pu | 0.069 | 0.047 | 1.475 | 62 | 0.145 |
| L Pu (A) → L vlPFC | 0.042 | 0.037 | 1.149 | 62 | 0.255 |
| L Pu (A) → R vlPFC | -0.027 | 0.045 | -0.604 | 62 | 0.548 |
| L Pu (B) → R FPC | -0.039 | 0.032 | -1.222 | 62 | 0.226 |
| L Pu (B) → L Pu (A) | -0.104 | 0.045 | -2.329 | 62 | 0.023 |
| L Pu (B) → R Pu | -0.056 | 0.037 | -1.507 | 62 | 0.137 |
| L Pu (B) → L vlPFC | -0.025 | 0.038 | -0.670 | 62 | 0.505 |
| L Pu (B) → R vlPFC | -0.020 | 0.038 | -0.541 | 62 | 0.591 |
| R Pu → R FPC | -0.037 | 0.046 | -0.807 | 62 | 0.423 |
| R Pu → L Pu (A) | 0.018 | 0.046 | 0.387 | 62 | 0.700 |
| R Pu → L Pu (B) | 0.033 | 0.043 | 0.771 | 62 | 0.444 |
| R Pu → L vlPFC | 0.016 | 0.037 | 0.443 | 62 | 0.659 |
| R Pu → R vlPFC | 0.040 | 0.043 | 0.918 | 62 | 0.362 |
| L vlPFC → R FPC | -0.015 | 0.045 | -0.321 | 62 | 0.749 |
| L vlPFC → L Pu (B) | 0.007 | 0.048 | 0.139 | 62 | 0.890 |
| L vlPFC → R Pu | -0.037 | 0.046 | -0.806 | 62 | 0.424 |
| L vlPFC → R vlPFC | -0.001 | 0.040 | -0.033 | 62 | 0.974 |
| R vlPFC → R FPC | 0.023 | 0.040 | 0.578 | 62 | 0.566 |
| R vlPFC → L Pu (A) | 0.003 | 0.040 | 0.070 | 62 | 0.945 |
| R vlPFC → R Pu | -0.075 | 0.053 | -1.426 | 62 | 0.159 |
| R vlPFC → L vlPFC | -0.002 | 0.037 | -0.052 | 62 | 0.959 |
| *BDD* | *β_Group_* | SE | *t* | *df* | *p_uncorrected_* |
| L vlPFC → L Pu (A) | -0.045 | 0.028 | -1.595 | 32 | 0.121 |
| R vlPFC → L Pu (B) | 0.006 | 0.026 | 0.218 | 32 | 0.829 |
| R FPC → R Pu | -0.013 | 0.029 | -0.428 | 32 | 0.672 |
| L SMA → L Pu (A) | 0.019 | 0.023 | 0.800 | 32 | 0.430 |
| R SMA → R Pu | 0.023 | 0.031 | 0.720 | 32 | 0.477 |
| L caudate → OFC | -0.006 | 0.028 | -0.220 | 32 | 0.827 |
| R caudate → OFC | 0.018 | 0.025 | 0.715 | 32 | 0.480 |
| R FPC → L Pu (A) | 0.014 | 0.027 | 0.524 | 32 | 0.604 |
| R FPC → L Pu (B) | -0.010 | 0.026 | -0.387 | 32 | 0.701 |
| R FPC → L vlPFC | 0.003 | 0.025 | 0.115 | 32 | 0.909 |
| R FPC → R vlPFC | 0.007 | 0.029 | 0.259 | 32 | 0.797 |
| L Pu (A) → R FPC | -0.016 | 0.029 | -0.549 | 32 | 0.587 |
| L Pu (A) → L Pu (B) | 0.021 | 0.028 | 0.755 | 32 | 0.456 |
| L Pu (A) → R Pu | -0.006 | 0.026 | -0.213 | 32 | 0.833 |
| L Pu (A) → L vlPFC | 0.047 | 0.026 | 1.813 | 32 | 0.079 |
| L Pu (A) → R vlPFC | -0.018 | 0.035 | -0.512 | 32 | 0.612 |
| L Pu (B) → R FPC | -0.012 | 0.030 | -0.383 | 32 | 0.704 |
| L Pu (B) → L Pu (A) | 0.034 | 0.027 | 1.254 | 32 | 0.219 |
| L Pu (B) → R Pu | 0.053 | 0.027 | 1.969 | 32 | 0.058 |
| L Pu (B) → L vlPFC | -0.046 | 0.024 | -1.927 | 32 | 0.063 |
| L Pu (B) → R vlPFC | 0.039 | 0.022 | 1.782 | 32 | 0.084 |
| R Pu → R FPC | -0.019 | 0.028 | -0.688 | 32 | 0.496 |
| R Pu → L Pu (A) | 0.011 | 0.036 | 0.320 | 32 | 0.751 |
| R Pu → L Pu (B) | -0.070 | 0.026 | -2.749 | 32 | 0.010* |
| R Pu → L vlPFC | -0.012 | 0.027 | -0.440 | 32 | 0.663 |
| R Pu → R vlPFC | -0.021 | 0.028 | -0.769 | 32 | 0.448 |
| L vlPFC → R FPC | 0.0002 | 0.029 | 0.008 | 32 | 0.994 |
| L vlPFC → L Pu (B) | 0.047 | 0.032 | 1.474 | 32 | 0.150 |
| L vlPFC → R Pu | 0.015 | 0.032 | 0.464 | 32 | 0.646 |
| L vlPFC → R vlPFC | 0.007 | 0.031 | 0.208 | 32 | 0.837 |
| R vlPFC → R FPC | 0.001 | 0.029 | 0.022 | 32 | 0.983 |
| R vlPFC → L Pu (A) | 0.062 | 0.025 | 2.454 | 32 | 0.020* |
| R vlPFC → R Pu | 0.047 | 0.026 | 1.812 | 32 | 0.080 |
| R vlPFC → L vlPFC | 0.048 | 0.029 | 1.680 | 32 | 0.103 |

**Supplemental Table 2.** Results from “post-hoc” multivariate linear regression models estimating the effect of group on mean dynamic effective connectivity. Each participant group was compared with their matched set of healthy controls. Displayed are the statistics for the effect of group; other variables in these models included age, gender and in-scanner motion (DVARS). For completeness, statistics for all 34 connections are displayed; the seven connections of interest are highlighted above the dashed line. *p*-values reported in the main text are corrected for multiple comparisons using a Bonferroni correction; all *p*-values reported in this table are uncorrected. OCD = obsessive compulsive disorder, BDD = body dysmorphic disorder, vlPFC = ventrolateral prefrontal cortex, Pu = posterolateral putamen, FPC = frontal polar cortex, SMA = supplemental motor area, OFC = orbitofrontal cortex. *Indicates statistical significance at *p* < 0.05 (uncorrected).

| *OCD* | *β* | SE | *t* | *df* | *p_uncorrected_* | *r* [95% CI] |
| --- | --- | --- | --- | --- | --- | --- |
| Intercept | 24.454 | 0.685 | 35.690 | 39 | < 0.001 | -- |
| L vlPFC → L Pu | 12.157 | 4.746 | 2.561 | 39 | **0.014*** | 0.328 [0.030, 0.572]* |
| R vlPFC → L Pu | 5.989 | 3.776 | 1.586 | 39 | 0.121 | -- |
| R FPC → R Pu | 2.654 | 4.345 | 0.611 | 39 | 0.545 | -- |
| Intercept | 24.596 | 0.710 | 34.631 | 40 | < 0.001 | -- |
| L SMA → L Pu | -6.084 | 4.886 | -1.245 | 40 | 0.220 | -- |
| R SMA → R Pu | 3.579 | 4.102 | 0.873 | 40 | 0.388 | -- |
| Intercept | 24.271 | 0.724 | 33.526 | 40 | < 0.001 | -- |
| R Caud → OFC | -7.068 | 4.906 | -1.441 | 40 | 0.157 | -- |
| L Caud → OFC | -7.206 | 4.340 | -1.660 | 40 | 0.105 | -- |
| *BDD* | *β* | SE | *t* | *df* | *p_uncorrected_* | *r* [95% CI] |
| Intercept | 22.597 | 0.855 | 26.425 | 17 | < 0.001 | -- |
| L vlPFC → L Pu | 23.542 | 14.869 | 1.583 | 17 | 0.135 | 0.456 [0.030, 0.742]* |
| R vlPFC → L Pu | -17.258 | 12.925 | -1.335 | 17 | 0.199 | -- |
| R FPC → R Pu | -2.720 | 11.806 | -0.230 | 17 | 0.821 | -- |
| Intercept | 27.121 | 1.028 | 26.370 | 18 | < 0.001 | -- |
| L SMA → L Pu | 16.480 | 15.254 | 1.080 | 18 | 0.294 | -- |
| R SMA → R Pu | 3.666 | 14.023 | 0.261 | 18 | 0.797 | -- |
| Intercept | 27.094 | 0.932 | 29.070 | 18 | < 0.001 | -- |
| R Caud → OFC | -20.145 | 13.505 | -1.492 | 18 | 0.153 | -- |
| L Caud → OFC | -14.410 | 12.893 | -1.118 | 18 | 0.278 | -- |

**Supplemental Table 3.** Multivariate linear regression models associating mean dynamic effective connectivity with Yale-Brown Obsessive Compulsive Scale (YBOCS) scores. Note that for participants with body dysmorphic disorder (BDD) we used items 1-10 of the version of the YBOCS adapted for BDD (BDD-YBOCS), to match the structure of the YBOCS. In additional to model coefficients and related statistics, displayed are univariate Pearson correlation coefficients and associated 95% confidence intervals for the left ventrolateral prefrontal cortex to the left posterior putamen connection, since it was significantly associated with YBOCS in the OCD dataset. *p*-values reported in the main text are corrected for multiple comparisons using a Bonferroni correction; all *p*-values reported in this table are uncorrected. OCD = obsessive compulsive disorder, BDD = body dysmorphic disorder, *r* = Pearson’s correlation, vlPFC = ventrolateral prefrontal cortex, Pu = posterolateral putamen, FPC = frontal polar cortex, SMA = supplemental motor area, Caud = caudate, OFC = orbitofrontal cortex. *Indicates statistical significance at *p* < 0.05.

| *OCD* | *r* [95% CI] | *p_uncorrected_* |
| --- | --- | --- |
| L vlPFC → L Pu (A) | 0.328 [0.030 0.572] | **0.032*** |
| R vlPFC → L Pu (B) | 0.182 [-0.125 0.457] | 0.242 |
| R FPC → R Pu | 0.017 [-0.285 0.316] | 0.913 |
| L SMA → L Pu (A) | -0.218 [-0.486 0.088] | 0.161 |
| R SMA → R Pu | 0.170 [-0.137 0.448] | 0.275 |
| L caudate → OFC | -0.157 [-0.437 0.150] | 0.314 |
| R caudate → OFC | -0.095 [-0.384 0.211] | 0.546 |
| R FPC → L Pu (A) | -0.039 [-0.336 0.264] | 0.802 |
| R FPC → L Pu (B) | 0.040 [-0.264 0.336] | 0.801 |
| R FPC → L vlPFC | 0.034 [-0.269 0.331] | 0.829 |
| R FPC → R vlPFC | 0.078 [-0.228 0.369] | 0.620 |
| L Pu (A) → R FPC | -0.160 [-0.440 0.147] | 0.304 |
| L Pu (A) → L Pu (B) | -0.032 [-0.329 0.271] | 0.839 |
| L Pu (A) → R Pu | -0.392 [-0.619 -0.104] | 0.009* |
| L Pu (A) → L vlPFC | -0.224 [-0.491 0.082] | 0.149 |
| L Pu (A) → R vlPFC | 0.045 [-0.259 0.341] | 0.775 |
| L Pu (B) → R FPC | -0.111 [-0.398 0.196] | 0.480 |
| L Pu (B) → L Pu (A) | 0.164 [-0.143 0.443] | 0.292 |
| L Pu (B) → R Pu | 0.193 [-0.114 0.466] | 0.216 |
| L Pu (B) → L vlPFC | 0.233 [-0.072 0.499] | 0.132 |
| L Pu (B) → R vlPFC | 0.015 [-0.287 0.314] | 0.925 |
| R Pu → R FPC | 0.031 [-0.272 0.328] | 0.844 |
| R Pu → L Pu (A) | 0.258 [-0.046 0.518] | 0.095 |
| R Pu → L Pu (B) | 0.047 [-0.257 0.342] | 0.766 |
| R Pu → L vlPFC | -0.006 [-0.306 0.295] | 0.971 |
| R Pu → R vlPFC | 0.154 [-0.154 0.434] | 0.325 |
| L vlPFC → R FPC | 0.022 [-0.280 0.320] | 0.889 |
| L vlPFC → L Pu (B) | 0.121 [-0.186 0.407] | 0.440 |
| L vlPFC → R Pu | 0.001 [-0.299 0.301] | 0.994 |
| L vlPFC → R vlPFC | 0.130 [-0.177 0.414] | 0.406 |
| R vlPFC → R FPC | -0.107 [-0.395 0.199] | 0.493 |
| R vlPFC → L Pu (A) | 0.013 [-0.289 0.312] | 0.935 |
| R vlPFC → R Pu | -0.144 [-0.426 0.164] | 0.358 |
| R vlPFC → L vlPFC | 0.087 [-0.219 0.377] | 0.580 |
| *BDD* | *r* [95% CI] | *p_uncorrected_* |
| L vlPFC → L Pu (A) | 0.456 [0.030 0.742] | **0.038*** |
| R vlPFC → L Pu (B) | -0.396 [-0.707 0.043] | 0.075 |
| R FPC → R Pu | -0.100 [-0.510 0.346] | 0.665 |
| L SMA → L Pu (A) | 0.282 [-0.171 0.636] | 0.216 |
| R SMA → R Pu | 0.153 [-0.298 0.549] | 0.507 |
| L caudate → OFC | -0.316 [-0.658 0.133] | 0.162 |
| R caudate → OFC | -0.379 [-0.697 0.063] | 0.090 |
| R FPC → L Pu (A) | 0.603 [0.231 0.821] | 0.004* |
| R FPC → L Pu (B) | 0.372 [-0.071 0.693] | 0.096 |
| R FPC → L vlPFC | -0.393 [-0.705 0.046] | 0.078 |
| R FPC → R vlPFC | -0.220 [-0.595 0.234] | 0.338 |
| L Pu (A) → R FPC | -0.325 [-0.664 0.124] | 0.150 |
| L Pu (A) → L Pu (B) | -0.457 [-0.742 -0.032] | 0.037* |
| L Pu (A) → R Pu | -0.126 [-0.529 0.324] | 0.588 |
| L Pu (A) → L vlPFC | 0.020 [-0.415 0.448] | 0.932 |
| L Pu (A) → R vlPFC | 0.169 [-0.284 0.560] | 0.465 |
| L Pu (B) → R FPC | -0.013 [-0.443 0.421] | 0.954 |
| L Pu (B) → L Pu (A) | 0.181 [-0.272 0.568] | 0.433 |
| L Pu (B) → R Pu | -0.207 [-0.587 0.246] | 0.367 |
| L Pu (B) → L vlPFC | -0.270 [-0.629 0.183] | 0.236 |
| L Pu (B) → R vlPFC | -0.353 [-0.681 0.093] | 0.116 |
| R Pu → R FPC | -0.028 [-0.454 0.409] | 0.905 |
| R Pu → L Pu (A) | 0.189 [-0.264 0.574] | 0.412 |
| R Pu → L Pu (B) | 0.039 [-0.399 0.463] | 0.865 |
| R Pu → L vlPFC | -0.301 [-0.648 0.15] | 0.185 |
| R Pu → R vlPFC | 0.062 [-0.380 0.481] | 0.789 |
| L vlPFC → R FPC | 0.135 [-0.315 0.535] | 0.561 |
| L vlPFC → L Pu (B) | 0.232 [-0.222 0.603] | 0.312 |
| L vlPFC → R Pu | 0.196 [-0.257 0.579] | 0.393 |
| L vlPFC → R vlPFC | 0.119 [-0.330 0.524] | 0.608 |
| R vlPFC → R FPC | 0.119 [-0.329 0.524] | 0.606 |
| R vlPFC → L Pu (A) | -0.321 [-0.661 0.129] | 0.156 |
| R vlPFC → R Pu | -0.201 [-0.582 0.252] | 0.382 |
| R vlPFC → L vlPFC | -0.084 [-0.498 0.361] | 0.717 |

**Supplemental Table 4.** Bivariate Pearson’s correlations (as well as associated 95% confidence intervals and *p*-values) between Yale-Brown Obsessive Compulsive Scale (YBOCS) scores and mean dynamic effective connectivity for all 34 connections. Note that for participants with body dysmorphic disorder (BDD), we used items 1-10 of the version of the YBOCS adapted for BDD (BDD-YBOCS), to match the structure of the YBOCS. Highlighted above the dashed line for both datasets are the seven connections of interest. All *p*-values reported in this table are uncorrected. OCD = obsessive compulsive disorder, BDD = body dysmorphic disorder, *r* = Pearson’s correlation, vlPFC = ventrolateral prefrontal cortex, Pu = posterolateral putamen, FPC = frontal polar cortex. *Indicates statistical significance at *p* < 0.05.


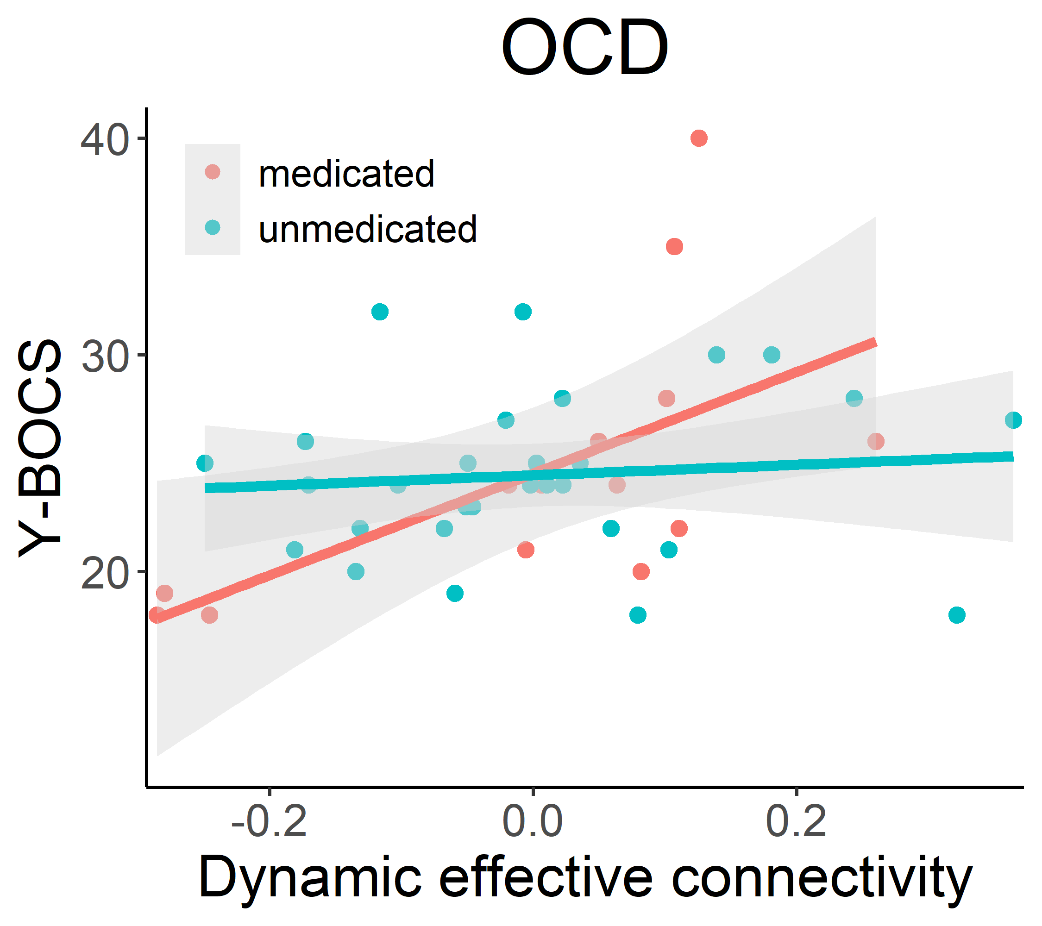


**Supplemental Figure 1.** Correlation of mean dynamic effective connectivity of the left ventrolateral prefrontal cortex → left posterolateral putamen connection with Yale-Brown Obsessive Compulsive Scale (YBOCS), separated by medication status. Medicated (any psychotropic medication) participants exhibited a positive correlation with YBOCS, while unmedicated participants did not exhibit a significant correlation. Gray ribbons indicate 95% confidence intervals. OCD = obsessive compulsive disorder, YBOCS = Yale-Brown Obsessive Compulsive Scale.


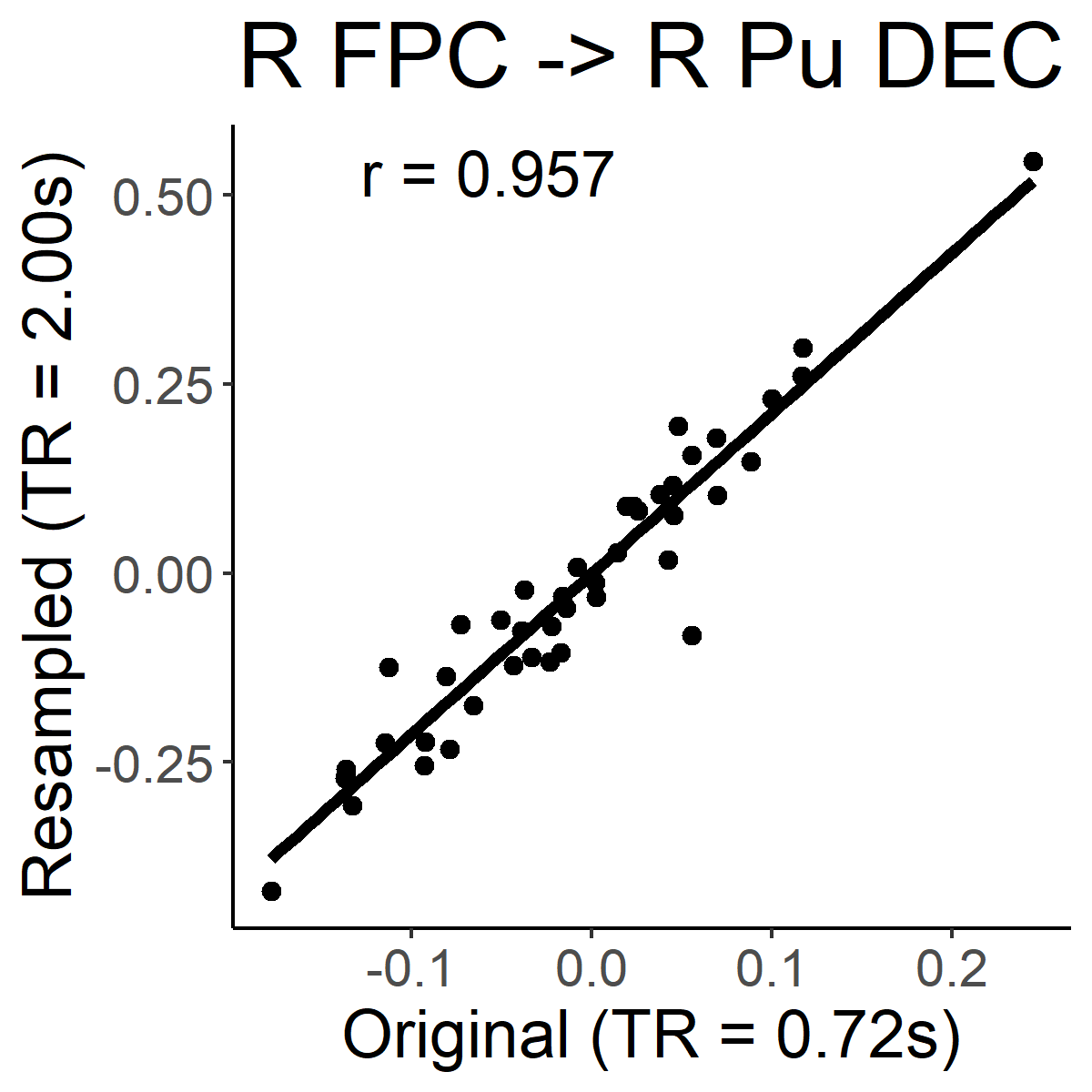

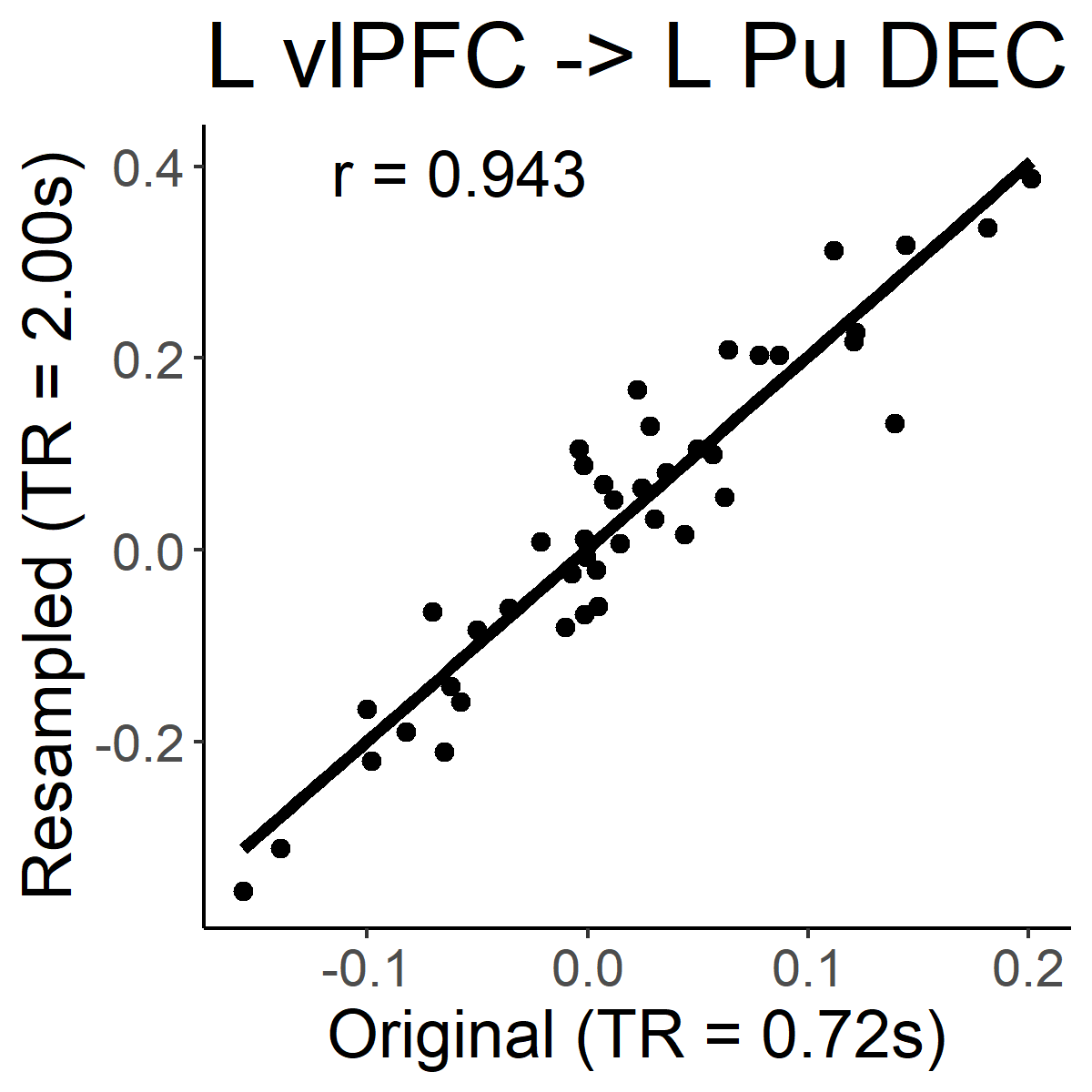

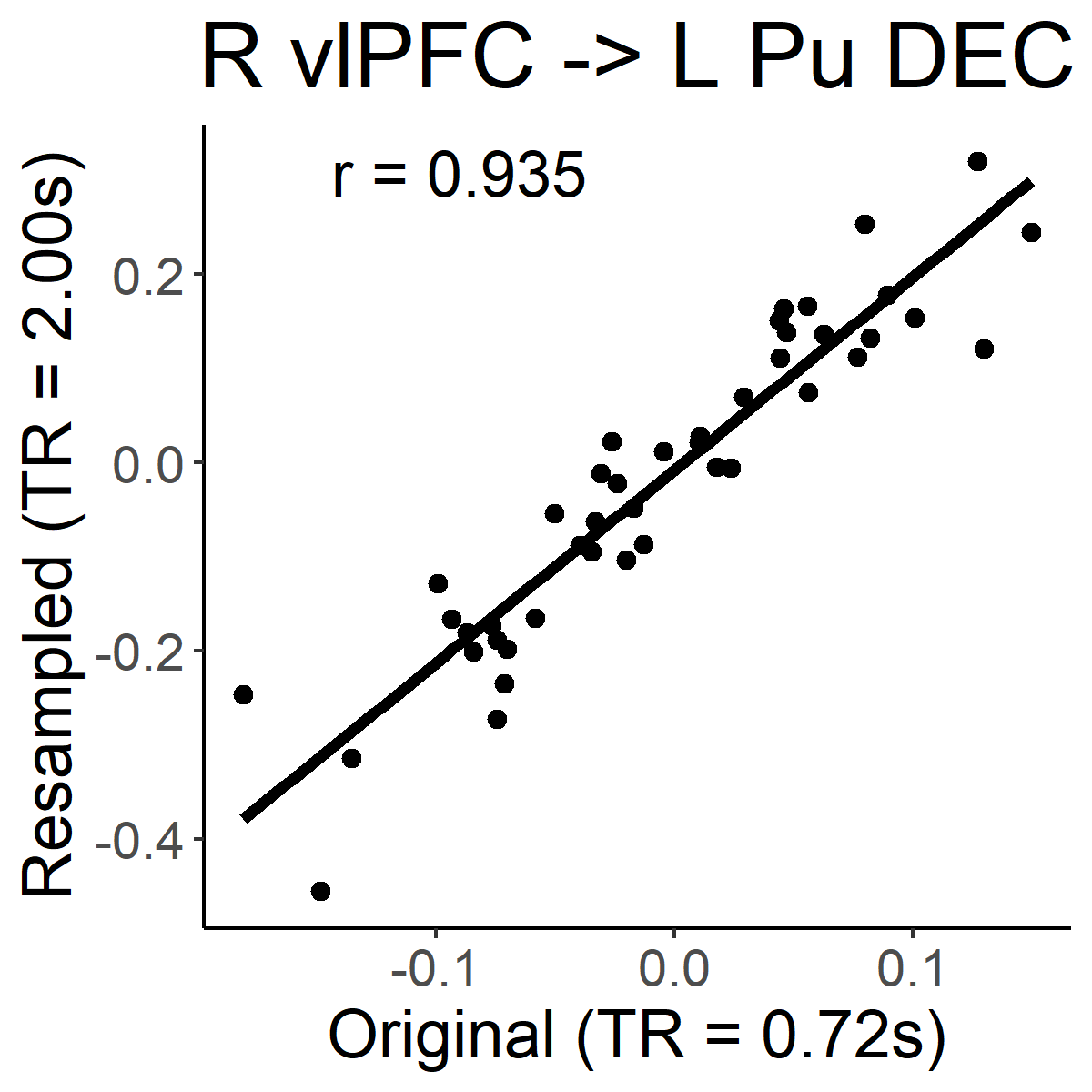


**Supplemental Figure 2.** Scatterplots displaying correlation between dynamic effective connectivity (DEC) estimates before (“Original”) and after (“Resampled”) resampling of repetition time (TR) of the body dysmorphic disorder dataset. Displayed are the three connections that were analyzed in the main text. FPC = frontopolar cortex, vlPFC = ventrolateral prefrontal cortex, Pu = posterolateral putamen.


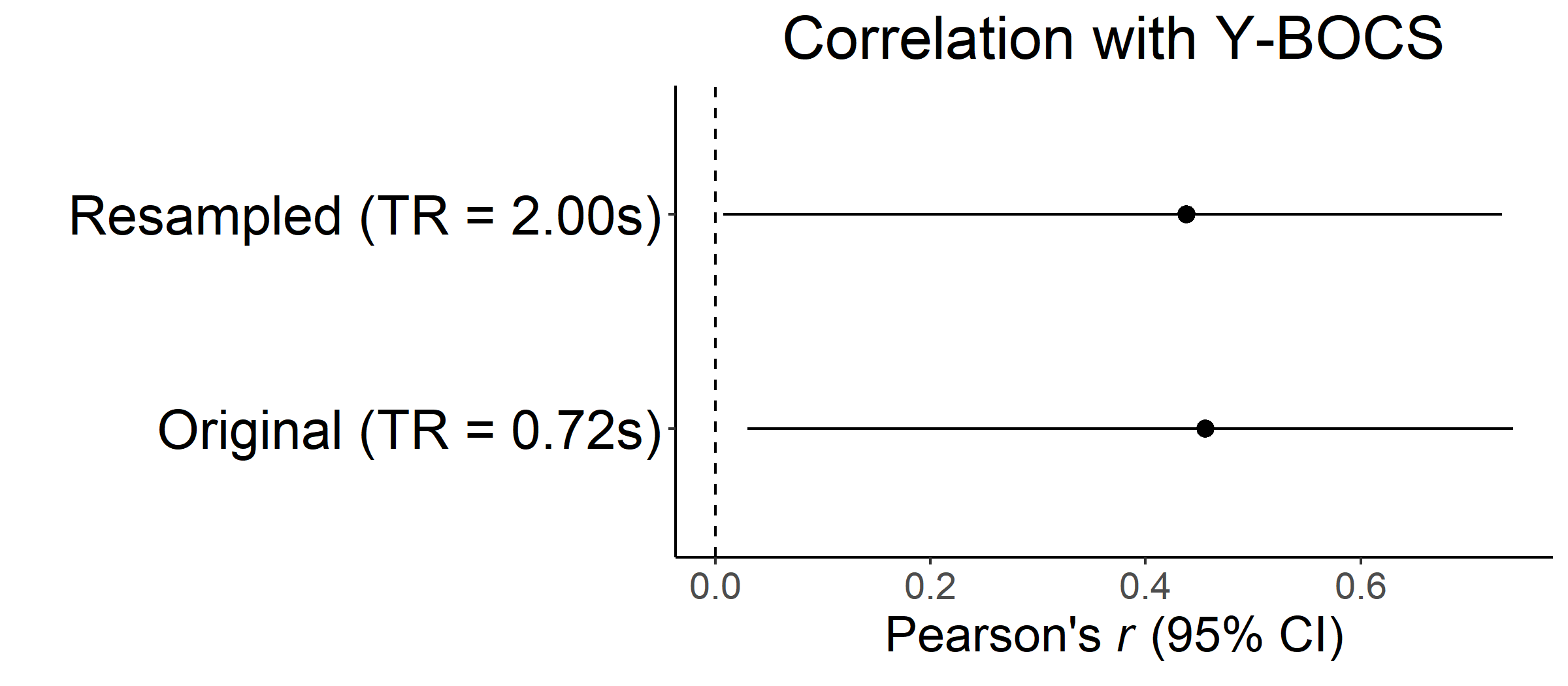


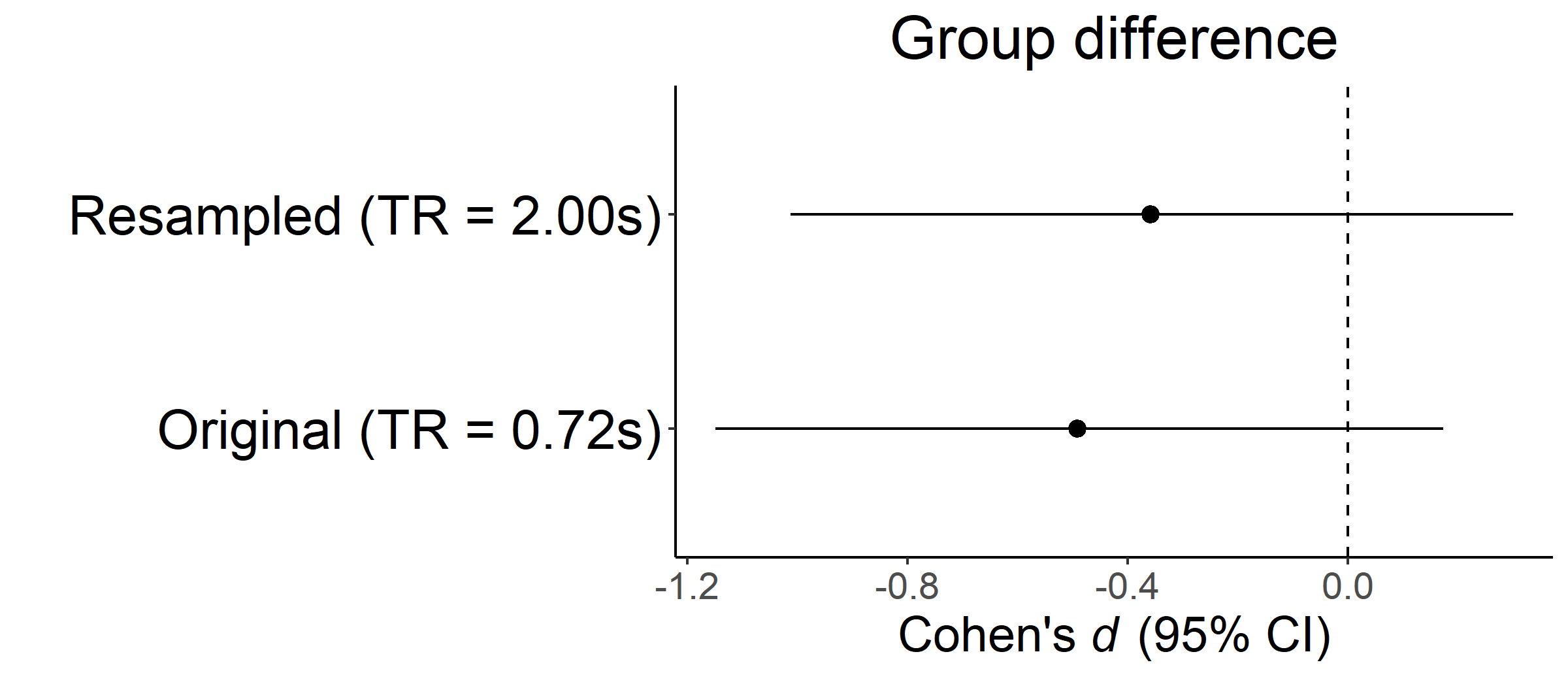


**Supplemental Figure 3.** Forest plots of effect sizes for mean dynamic effective connectivity (DEC) of the left ventrolateral prefrontal cortex → left posterolateral putamen connection for the body dysmorphic disorder (BDD) dataset, before (“Original”) and after (“Resampled”) resampling of repetition time (TR). Plotted are point estimates of effect sizes and 95% confidence intervals. Upper plot displays Pearson’s correlations between mean DEC and the BDD-Y-BOCS (Yale-Brown Obsessive Compulsive Scale). Lower plot displays Cohen’s *d* of the group difference in mean DEC between BDD participants and their healthy controls.

**
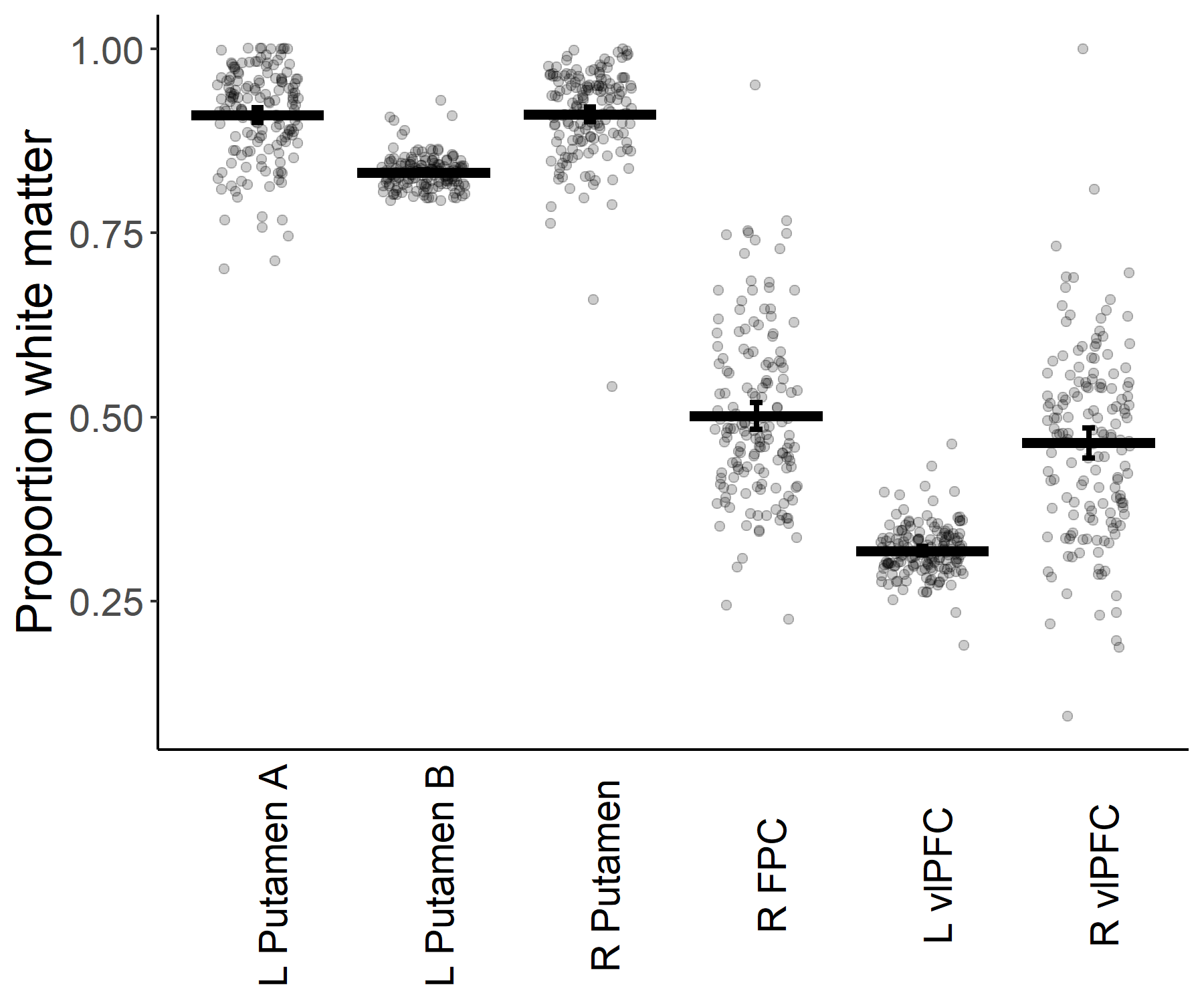
**

**Supplemental Figure 4.** Quantifications of percentage white matter for the six regions of interest, across all participants. Each dot represents a participant, and horizontal black lines indicate the group mean. Putamen seeds exhibit high percentages of white matter (see Limitations and Considerations subsection for discussion about evidence of “true”, neural activity-related signal from predominately white matter regions). FPC = frontal polar cortex, vlPFC = ventrolateral prefrontal cortex.

*Supplemental References*

[1] T. A. Brown, D. H. Barlow, and P. A. DiNardo, *Anxiety disorders interview schedule adult version: Client interview schedule.* Graywind Publications Incorporated, 1994.

[2] W. K. Goodman *et al.*, “The Yale-Brown Obsessive Compulsive Scale: I. Development, Use, and Reliability,” *Arch. Gen. Psychiatry*, vol. 46, no. 11, pp. 1006–1011, 1989.

[3] D. V. Sheehan, “The Mini-International Neuropsychiatric Interview (M.I.N.I.): The Development and Validation of a Structured Diagnostic Psychiatric Interview for DSM-IV and ICD-10,” *J Clin Psychiatry*, p. 12.

[4] R. G. Dufresne, K. A. Phillips, C. C. Vittorio, and C. S. Wilkel, “A Screening Questionnaire for Body Dysmorphic Disorder in a Cosmetic Dermatologic Surgery Practice,” *Dermatol Surg*, p. 6, 2001.

[5] O. Esteban *et al.*, “fMRIPrep: a robust preprocessing pipeline for functional MRI,” *Nat. Methods*, vol. 16, no. 1, pp. 111–116, Jan. 2019, doi: 10.1038/s41592-018-0235-4.

[6] K. Gorgolewski *et al.*, “Nipype: A Flexible, Lightweight and Extensible Neuroimaging Data Processing Framework in Python,” *Front. Neuroinformatics*, vol. 5, 2011, doi: 10.3389/fninf.2011.00013.

[7] N. J. Tustison *et al.*, “N4ITK: Improved N3 Bias Correction,” *IEEE Trans. Med. Imaging*, vol. 29, no. 6, pp. 1310–1320, Jun. 2010, doi: 10.1109/TMI.2010.2046908.

[8] V. Fonov, A. C. Evans, K. Botteron, C. R. Almli, R. C. McKinstry, and D. L. Collins, “Unbiased average age-appropriate atlases for pediatric studies,” *NeuroImage*, vol. 54, no. 1, pp. 313–327, Jan. 2011, doi: 10.1016/j.neuroimage.2010.07.033.

[9] B. Avants, C. Epstein, M. Grossman, and J. Gee, “Symmetric diffeomorphic image registration with cross-correlation: Evaluating automated labeling of elderly and neurodegenerative brain,” *Med. Image Anal.*, vol. 12, no. 1, pp. 26–41, Feb. 2008, doi: 10.1016/j.media.2007.06.004.

[10] R. W. Cox, “AFNI: Software for Analysis and Visualization of Functional Magnetic Resonance Neuroimages,” *Comput. Biomed. Res.*, vol. 29, no. 3, pp. 162–173, Jun. 1996, doi: 10.1006/cbmr.1996.0014.

[11] M. Jenkinson, P. Bannister, M. Brady, and S. Smith, “Improved Optimization for the Robust and Accurate Linear Registration and Motion Correction of Brain Images,” *NeuroImage*, vol. 17, no. 2, pp. 825–841, Oct. 2002, doi: 10.1006/nimg.2002.1132.

[12] J. Huntenburg and L. Str, “Evaluating nonlinear coregistration of BOLD EPI and T1w images,” p. 29, 2014.

[13] S. Wang, D. J. Peterson, J. C. Gatenby, W. Li, T. J. Grabowski, and T. M. Madhyastha, “Evaluation of Field Map and Nonlinear Registration Methods for Correction of Susceptibility Artifacts in Diffusion MRI,” *Front. Neuroinformatics*, vol. 11, Feb. 2017, doi: 10.3389/fninf.2017.00017.

[14] J. M. Treiber *et al.*, “Characterization and Correction of Geometric Distortions in 814 Diffusion Weighted Images,” *PLOS ONE*, vol. 11, no. 3, p. e0152472, Mar. 2016, doi: 10.1371/journal.pone.0152472.

[15] D. N. Greve and B. Fischl, “Accurate and robust brain image alignment using boundary-based registration,” *NeuroImage*, vol. 48, no. 1, pp. 63–72, Oct. 2009, doi: 10.1016/j.neuroimage.2009.06.060.

[16] R. H. R. Pruim, M. Mennes, D. van Rooij, A. Llera, J. K. Buitelaar, and C. F. Beckmann, “ICA-AROMA: A robust ICA-based strategy for removing motion artifacts from fMRI data,” *NeuroImage*, vol. 112, pp. 267–277, May 2015, doi: 10.1016/j.neuroimage.2015.02.064.

[17] A. Abraham *et al.*, “Machine learning for neuroimaging with scikit-learn,” *Front. Neuroinformatics*, vol. 8, 2014, doi: 10.3389/fninf.2014.00014.
